# Biofilm removal capacity and titanium surface integrity in non‐abrasive versus abrasive peri‐implantitis cleaning interventions

**DOI:** 10.1002/jper.11371

**Published:** 2025-12-10

**Authors:** Marzieh S. Jazaeri, Danyal A. Siddiqui, Yi‐Wen C. Tsai, Kathryn Gabel, Zachary Lorenzana, Georgios A. Kotsakis

**Affiliations:** ^1^ Department of Oral Biology Rutgers School of Dental Medicine Newark New Jersey USA; ^2^ Department of Periodontics UT Health School of Dentistry San Antonio Texas USA; ^3^ Department of Periodontology School of Dentistry Tri‐Service General Hospital and National Defense Medical Center Taipei City Taiwan; ^4^ Dedman College of Humanities and Sciences Southern Methodist University Dallas Texas USA

**Keywords:** biofilms, decontamination, fibroblasts, peri‐implantitis, surface properties, titanium

## Abstract

**Background:**

Current peri‐implantitis treatment methods are modeled after dental cleaning modalities like abrasive surface cleaning. However, mechanical abrasive cleaning not only inadequately removes implant biofilms but also compromises implant surface integrity with adverse biological effects. The goal of this study was to evaluate a non‐abrasive waterjet implant cleaning method to remove biofilm while preserving titanium surface and maintaining its cytocompatibility.

**Methods:**

Dental plaque‐derived multispecies biofilms were cultured on acid‐etched titanium disks. Biofilm was removed using either mechanical contact abrasive implant cleaning (titanium brush or curette) or a non‐contact waterjet irrigator in continuous or pulsed flow setting. Uncontaminated and untreated disks served as negative and positive controls, respectively. Bacterial viability post‐treatment was assessed by agar plating and live‐dead imaging. Titanium surface integrity was studied by scanning electron microscopy and optical profilometry. Host tissue compatibility was evaluated by human gingival fibroblast proliferation on titanium surface post‐cleaning.

**Results:**

Non‐contact waterjet irrigation significantly reduced viable bacterial counts by ≥90.9% (∼100‐fold) on titanium surface versus abrasively cleaned and untreated biofilm groups (all *p* < 0.05). Waterjet treatment maintained titanium surface integrity and roughness similar to pristine titanium. In contrast, abrasive cleaning damaged the microrough titanium surface and left viable bacterial residues. Fibroblast viability was restored (∼76.8%) on waterjet‐treated titanium to levels comparable to sterile control (*p* > 0.05), whereas titanium brush‐ or curette‐treated surfaces had significantly lower levels post‐cleaning (all *p* < 0.05).

**Conclusions:**

Non‐abrasive waterjet cleaning is a superior method for the clinical treatment of peri‐implantitis biofilms versus mechanical abrasive cleaning while maintaining titanium implant surface properties necessary for reintegration with peri‐implant tissue.

**Plain Language Summary:**

Dental implant infections are usually cleaned by scrubbing the implant surface to remove attached bacteria. However, this mode of cleaning can scratch the implant surface and produce tiny pieces of wear or particles which can be toxic and cause the implant to fail. In this study, a new cleaning method using a fast‐flowing stream of water, called waterjet cleaning, was tested. The waterjet cleaning was able to remove most bacteria from the implant material, while cleaning by scrubbing left small spots of bacteria on the surface. Additionally, waterjet‐cleaned surfaces looked like the original implant material surface, while scrubbing‐cleaned surfaces had pieces missing from the surface, which affected human gum tissue cells attachment. Waterjet cleaning is a favorable method to clean dental implant infections without damaging the implant and allow for human tissue to recover and reconnect with the dental implant.

## 1 INTRODUCTION

Despite the high success rate of dental implants, peri‐implantitis remains a major factor contributing to dental implant failure, with an estimated $2.3B spent in 2024 for its treatment and management.^1^ Between 10% and 20% of dental implants are affected by peri‐implantitis, corresponding to over 350,000 of implants placed annually.^2^, ^3^ Peri‐implantitis is an inflammatory lesion of the mucosa surrounding the implant, with progressive loss of supporting peri‐implant bone.^4^ Peri‐implantitis begins with bacterial infiltration of the peri‐implant sulcus, leading to biofilm formation rich in gram‐negative anaerobes and triggering activation of innate and adaptive immune responses.^5^, ^6^ The disease progresses from reversible peri‐implant mucositis to irreversible peri‐implantitis, marked by soft tissue damage, bone resorption, and potential implant failure.^5^, ^6^ Notably, these peri‐implant pathogenic bacteria are different than the traditional red complex bacteria and present a unique implant‐specific microniche that is highly resistant to current mechanical and pharmacological antimicrobial interventions.

Current peri‐implantitis treatments target biofilm removal through mechanical debridement with curettes and brushes, antibiotics, chemical detoxification, laser irradiation, photodynamic therapy, air abrasion, or even implantoplasty.^7^, ^8^, ^9^ Despite these aggressive interventions, peri‐implantitis recurrence occurs in up to 65.2% of implants post‐treatment.^10^ Furthermore, abrasive cleaning methods such as scaling and ultrasonic treatment were shown to alter the morphology of the implant surface and generate titanium particles.^11^, ^12^, ^13^ The presence of implant‐derived titanium particles is strongly associated with peri‐implantitis in humans, and mechanistic data suggest that these titanium particles amplify pro‐inflammatory responses to plaque biofilms.^14^, ^15^ Titanium particles have been shown to promote macrophage polarization in vitro and secretion of inflammatory cytokines causing subsequent bone loss in mice.^16^, ^17^ Thus, the long‐term accumulation of dissolution debris due to abrasive cleaning‐mediated damage to the protective titanium passivation layer may compromise the reintegration of the titanium surface with peri‐implant tissues.^18^, ^19^, ^20^, ^21^

Ideally, successful peri‐implantitis treatment occurs through (i) removing the majority of the biofilm, (ii) preserving titanium implant surface integrity, and (iii) maintaining cytocompatibility toward host tissue. However, the outcome of mechanical abrasive implant cleaning conflicts with these goals of peri‐implantitis treatment. That is, abrasive cleaning of the implant surface can accomplish partial biofilm removal but simultaneously damage the surface and compromise cytocompatibility. For example, an experimental study showed that abrasive and chemical implant surface cleaning using a titanium brush, with or without the adjunctive application of 1% sodium hypochlorite (NaOCl) and 0.2% chlorhexidine (CHX), resulted in fibrous encapsulation of the treated implant surfaces instead of osseointegration.^22^ On the other hand, non‐contact non‐abrasive cleaning methods like air abrasion can preserve the implant surface topography during cleaning; however, use of air abrasion around implants comes with the major health risk of emphysema even when used non‐surgically.^23^ Recently, a paradigm shift has occurred based on classification of cleaning aids into contact versus non‐contact with contact mechanical cleaning aids being further subcategorized into abrasive versus non‐abrasive.^24^ With the introduction of non‐contact cleaning aids such as pressurized irrigation, the opportunity exists to maximize biofilm surface removal without imparting damage to the biomaterial surface. The goal of the present study is to examine biofilm removal capacity, biomaterial surface integrity, and cytocompatibility of a non‐contact implant cleaning method using pressurized irrigation versus established contact mechanical cleaning aids.

## 2 MATERIALS AND METHODS

### 2.1 Specimen preparation

Grade 5 titanium (Ti) alloy (Ti‐6Al‐4 V) disks (Ø 10 × 2 mm) were individually etched in 1 mL of 34% sulfuric acid and 14% hydrochloric acid for 2 h in glass test tubes in an incubating shaker* at 600 rpm and 80°C to simulate clinical acid‐etched implant surfaces.^13^ Disks were cleaned post‐etching by ultrasonication in deionized water for 20 min thrice, sterilized via sequential ultrasonication in acetone, deionized water, and ethanol, and dried in an oven at 60°C prior to testing.

### 2.2 Multispecies bacterial culture

An ex vivo multispecies plaque sample was obtained from a patient with active peri‐implantitis in accordance with an Internal Review Board (IRB) approved protocol (IRB #: Pro2023001684). The sample was immediately cryopreserved in medium with 20% glycerol at −80°C and later revived anaerobically at 37°C in SHI broth medium per established protocol that supports diverse growth of oral microbiota from gram‐negative‐rich, site‐specific subgingival plaque samples.^25^ Briefly, 50 µL of −80°C plaque‐derived stock was inoculated in 5 mL of SHI broth and cultured in an anaerobic chamber (10% CO_2_, 10% H_2_, 80% N_2_) for 24 h at 37°C. The optical density at 600 nm wavelength (OD600) of plaque‐derived culture was measured with a cell density meter†. Bacterial culture was diluted in fresh SHI medium to 5 × 10^7^ colony‐forming units (CFU)/mL based on OD600 reading. Ti disks in 24‐well plates were immersed in 1 mL of diluted bacterial suspension and incubated anaerobically at 37°C for 48 h to facilitate adherent, early biofilm formation. The culture medium was replenished after 24 h by replacing 500 µL of spent medium with 525 µL of fresh SHI medium with minimal biofilm disruption. For consistency in culture viability between experiments, CFU counts of viable, cultivable microbes in broth were determined by plating serial dilutions of cultures on tryptic soy agar supplemented with 10 mg/L hemin, 10 mg/L vitamin K, and 5% defibrinated sheep blood. Subsequently, the taxonomic composition of the inocula was verified via 16S rRNA sequencing (Table S1 in online *Journal of Periodontology*), which confirmed the retention of over 80 individual taxa, including well‐characterized peri‐implantitis genera such as *Fusobacterium, Porphoromonas*, and *Tannerella*.

### 2.3 Cleaning methods

Established abrasive implant cleaning was performed by scaling with a titanium curette or titanium brush and compared against an experimental pressurized water irrigation system using a continuous or pulsed water flow setting. All cleaning procedures were done by one trained individual and applied to the top and bottom disk surfaces. Negative control disks were incubated in uninoculated broth medium (no biofilm) while positive control samples had biofilms without any applied cleaning treatment.

Treatment by curette was performed using a Ti Gracey curette‡ with 30 strokes on each side of the disk by a single operator applying moderate pressure similar to that used for scaling implant abutments. Brush treatment was evaluated using a NiTi brush§ powered by a surgical motor and applied for 15 s at each side of the disk at a speed of 1200 rpm and a torque of 20 N•cm. During curette or brush treatment, 0.9% saline was applied to irrigate the surface while cleaning. To determine the most effective cleaning setting for the experimental pressurized waterjet cleaning instrument‖, three water flow rates (25, 50, or 75 mL/min) and three dispensing methods consisting of continuous mode, 3 pulses/s, or 6 pulses/s were assessed. Each disk was held on a meshed surface with tweezers to avoid movement and sprayed with saline through the instrument handpiece for 30 s per side. Waterjet cleaning was performed with smooth linear and circular motions. The nozzle was kept at an approximate distance of 2 cm from the disk at a 45° angle.

### 2.4 Evaluation of biofilm removal capacity

#### 2.4.1 Quantification of microbial viability

Each disk was immersed into 1 mL phosphate buffered saline (PBS) in a glass tube and sonicated for 5 min to detach adherent microbes. Ten‐fold serial dilutions were performed in PBS and plated on tryptic soy agar supplemented with 10 mg/L hemin, 10 mg/L vitamin K1, and 5% defibrinated sheep blood. CFU counts were evaluated 24‐ to 48‐h post‐treatment.

#### 2.4.2 Visualization of residual microbes on Ti surface

Treated samples were stained for 30 min with 10 µM SYTO9 and 60 µM propidium iodide to assess live and dead microbes, respectively. After staining, samples were rinsed thrice with deionized water and imaged using a super resolution microscope¶. The top surface of disk samples was imaged using a water immersion objective at 86x magnification. For semi‐quantification, residual biofilm on Ti disks post‐cleaning was stained with crystal violet solution (0.5% in 25% methanol) for 10 min and rinsed three times with deionized water to remove excessive stain. Stained biofilms were dissolved in 0.5 mL of 30% acetic acid with shaking at 200 rpm for 30 min, and 0.1 mL of solution per sample was transferred to a 96‐well plate for absorbance detection at 590 nm using a plate reader#.

### 2.5 Evaluation of titanium surface integrity

#### 2.5.1 Surface morphology and elemental analysis post‐treatment

A benchtop scanning electron microscope** was used to observe any alterations in the surface morphology of treated Ti disks versus control. Also, residual microbes and biofilm were detected after fixation, dehydration, and drying of samples. For fixation, samples were immersed in 1 mL of 2.5% glutaraldehyde in sodium cacodylate buffer, pH 7.4 for 30 min and then rinsed with deionized water twice. Dehydration was performed using an ascending ethanol series (1× for 10 min in 25%, 50%, and 75%, 2× for 5 min in 90%, and 3× for 5 min in 100%). Afterward, samples were dehydrated by sequential immersion in 33% and 67% hexamethyldisilazane (HMDS) in ethanol for 20 min before overnight drying in 100% HMDS. A conductive coating (8 nm gold) was applied to specimens prior to scanning electron microscopy (SEM) imaging using backscatter and/or secondary electron detection with a 15 kV beam. Surface elemental analysis was conducted using the same instrument equipped with energy‐dispersive x‐ray spectroscopy (EDS). The weight concentration of the elements on the surface was collected and compared among groups.

#### 2.5.2 Surface roughness analysis post‐treatment

An optical profilometer†† was used to measure mean surface roughness, Ra, post‐cleaning treatment. Three sample disks per group were examined, and three randomly selected areas (0.68 × 0.48 mm) at 10× magnification were scanned.

### 2.6 Evaluation of cytocompatibility

Human gingival fibroblasts (HGF‐1 cell line)‡‡ were cultured in Dulbecco's Modified Eagle Medium (DMEM) supplemented with 10% fetal bovine serum at 37°C in 5% CO_2_. Post‐cleaning, Ti sample disks were immediately autoclaved to neutralize live microbes. Fibroblasts were directly seeded in 50 µL drops on treated disks at concentrations of 10^4^ cells per disk in 12‐well plates and allowed to attach for 3 h. Two mL of culture medium was added, and fibroblasts were allowed to proliferate for 72 h. Afterward, fibroblasts on Ti disks were fixed with 4% paraformaldehyde, rinsed thrice in PBS, and stained for nuclei and F‐actin with 4′,6‐diamidino‐2‐phenylindole (DAPI)§§ and phalloidin conjugate‖‖ per manufacturer protocol. Fibroblast morphology was assessed using an inverted fluorescent microscope¶¶, and the number of fibroblast nuclei on Ti disks in three randomly selected areas at 20× magnification was counted using an image analysis software## to enumerate fibroblast proliferation.

### 2.7 Statistical analysis

Data analysis was performed with a scientific statistics program***. One‐way analysis of variance (ANOVA) was used to compare the effect of treatments, and Tukey's test for multiple comparisons. Statistical significance between groups was set at *α*  =  0.05.

## 3 RESULTS

### 3.1 Microbial biofilm removal efficacy

#### 3.1.1 Viable residual microbial count

Preliminary assessment of the waterjet cleaning was performed using different flow rates and pulsation settings to determine optimal operation settings. Irrigation flow between 40–55 mL/min reduced microbial CFU to the greatest extent (Figure S1 in online *Journal of Periodontology*). Subsequent tests using different pulsation settings showed that rates of 3 or 6 pulses/s were as effective (*p* > 0.05) as continuous irrigation in reducing microbial CFU (Figure S2 in online *Journal of Periodontology*). Two final settings of irrigation flow at 50 mL/min with continuous irrigation (C) or 6 pulses/s (P) were chosen to compare the efficacy of the waterjet with other abrasive cleaning methods.

**FIGURE 1:**
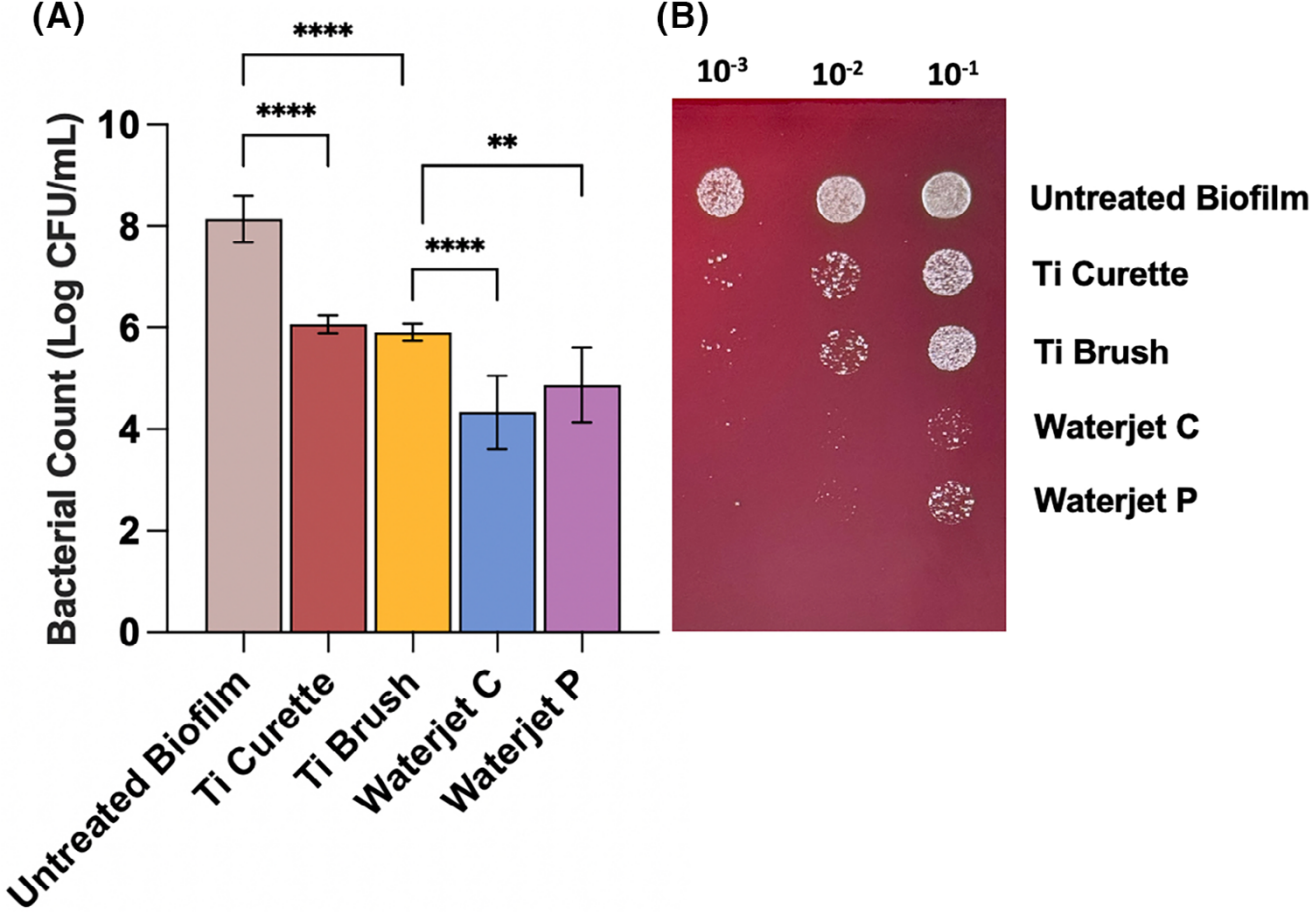
Comparison of microbial biofilm removal capacity on titanium (Ti) disks following various cleaning treatments: Ti curette, Ti brush, waterjet in continuous (C) or pulse (P) modes, and untreated biofilm control (*n* = 5). (A) Microbial count (log CFU/mL) for each treatment group. Statistical significance is indicated as *p* < 0.01 (**) and *p* < 0.0001 (****). (B) Representative images of serial dilutions (10^0^, 10^−1^, and 10^−2^) of microbial colonies plated on agar, depicting differences in residual microbial amount obtained from treated Ti surfaces. CFU, colony‐forming units.

All treatment modalities showed a significant reduction in CFU count compared with the untreated control group, though significant differences in effectiveness were noted among the test groups (Figure 1). Disks treated by curette or brush exhibited the smallest (∼100‐fold) decrease in viable microbial CFU count versus control, which were inferior to the waterjet groups. Both waterjet groups demonstrated an even greater reduction (∼1000–10,000‐fold vs.) in CFU count versus control, which was also significantly lower than curette and brush groups (*p* < 0.0001 and 0.01). The continuous and pulsed waterjet irrigation were 97.4% and 90.9% more effective in biofilm removal (∼100‐fold decrease), respectively, as compared to the Ti Brush group (*p* < 0.01). No significant difference was observed between waterjet continuous and pulse cleaning (*p* > 0.05).

#### 3.1.2 Viable residual biofilm

**FIGURE 2:**
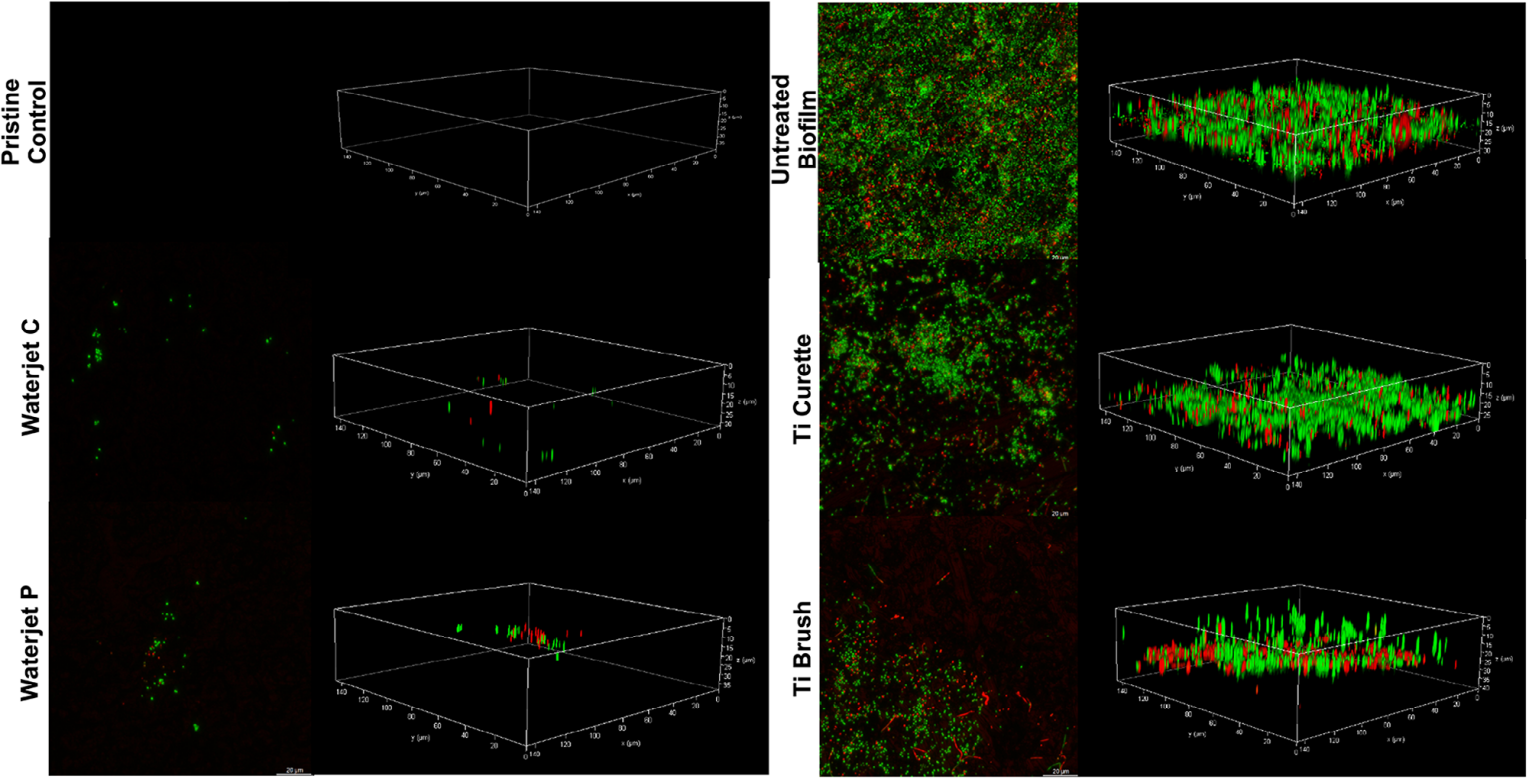
Representative 2D (left) and 3D (right) confocal images (20×) depicting fluorescently labeled live (green) and dead (red) bacteria within residual biofilm on Ti disks after cleaning treatment with Ti curette, Ti brush, or waterjet in continuous (C) or pulse (P) modes versus positive (untreated biofilm) and negative controls (pristine uncontaminated) for biofilm.

Confocal imaging of residual biofilm after cleaning treatment is depicted in Figure 2. All cleaning treatments reduced adherent biofilm coverage post‐treatment versus untreated control (Figure 2 upper right panel). The waterjet treatments resulted in the lowest observed amount of live microbes on Ti surface with near complete elimination of the 3D biofilm structure (Figure 2 middle and lower left panel). In contrast, multiple clusters or pockets of live (green) bacteria were still present on Ti disks treated with Ti curette or Ti brush. Notably, surfaces treated with Ti curette demonstrated an interrupted pattern of biofilm removal (Figure 2 middle right panel) with a few dark, void areas representing biofilm removal scattered within non‐cleaned areas with intact biofilm. This residual bacterial profile is suggestive of topical effects of abrasive biofilm removal where the cutting area of the curette meets the Ti surface. Similarly, disks treated with Ti brush contained a higher number of scattered colonies of viable microbes as compared to abraded areas that demonstrated biofilm removal (Figure 2 lower right panel). The areas of biofilm presence had biofilm thickness similar to untreated controls, suggesting limited surface access with titanium brush. Biofilm biomass quantification with crystal violet staining confirmed the trends noted in these results (Figure S3 in online *Journal of Periodontology*).

### 3.2 Maintenance of titanium surface integrity

#### 3.2.1 Surface morphology and elemental analysis post‐treatment

**FIGURE 3:**
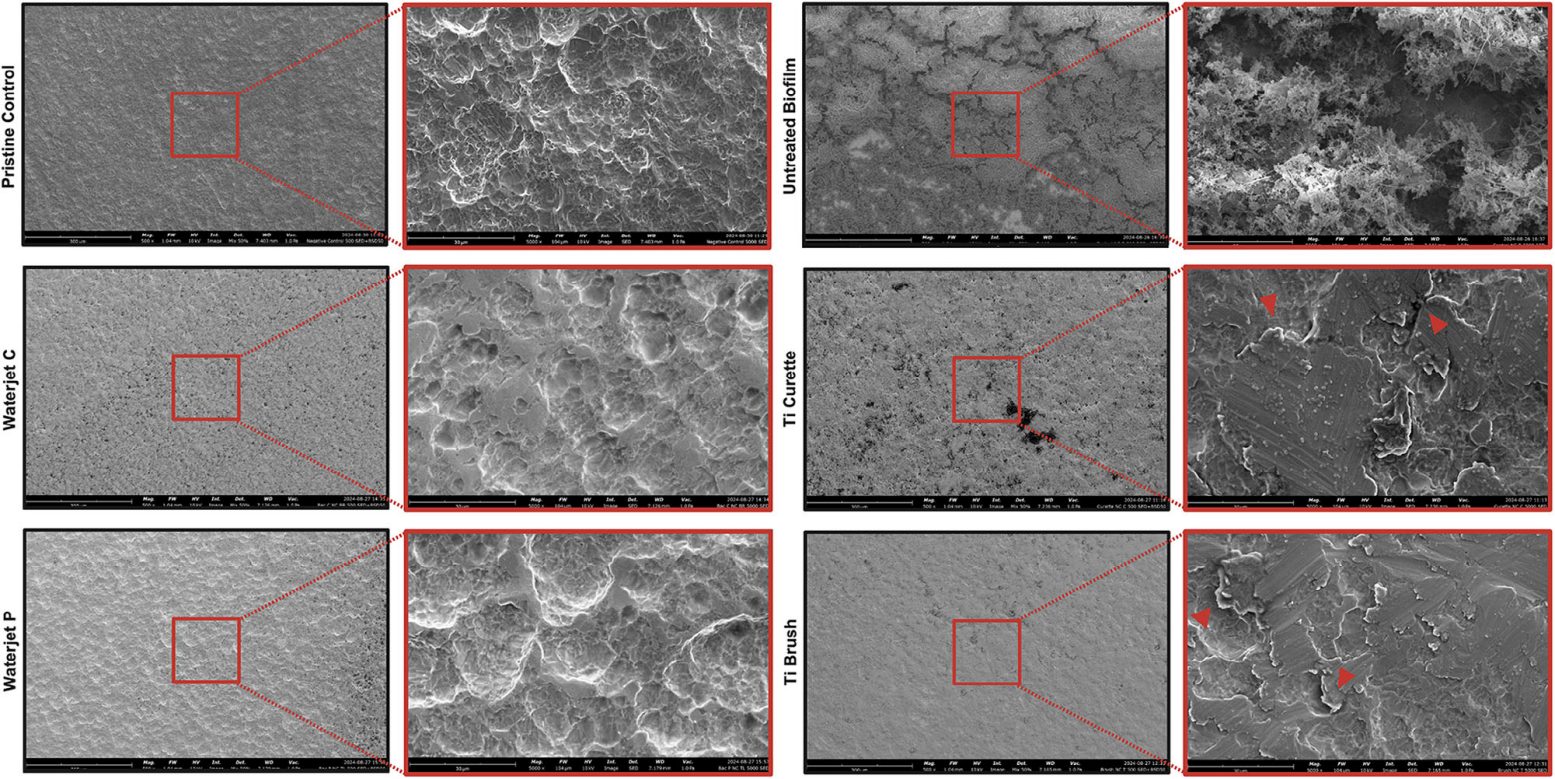
Representative SEM images at low magnification (left, 500×) with a higher magnification zoom‐in (right, 5000×) showing the surface morphology and the presence or reduction of biofilm on Ti disk surfaces following different cleaning treatments. Red arrows in Ti curette‐ and Ti brush‐treated samples indicate a delaminated Ti surface, suggesting potential mechanical damage due to abrasive mechanical cleaning techniques. Notably the EDS assesses the elemental composition at a depth > 500 nm suggesting that the abrasive interventions created considerable damage beyond the passivation layer. EDS, energy‐dispersive x‐ray spectroscopy; SEM, scanning electron microscope; Ti, titanium.

**FIGURE 4:**
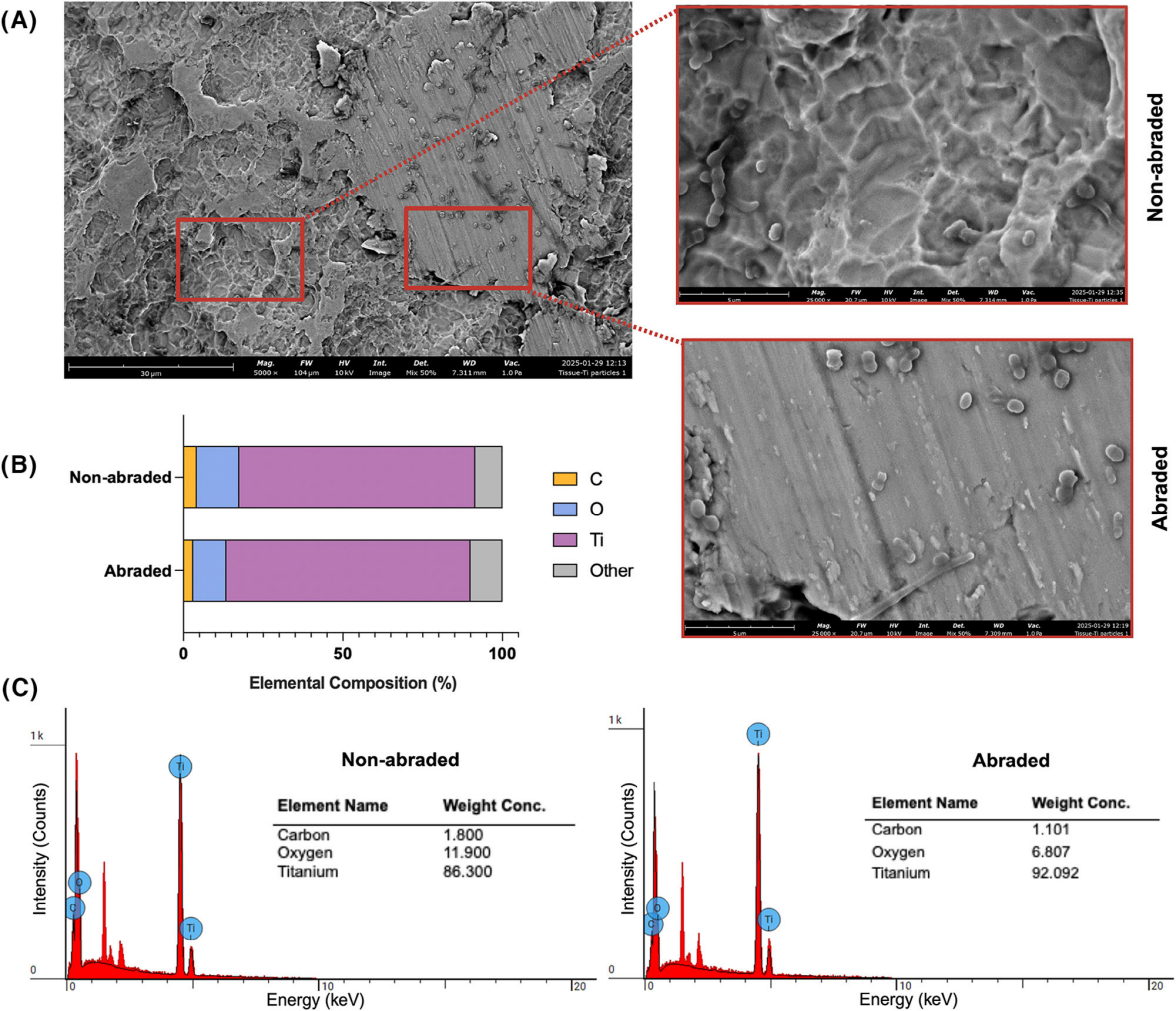
(A) Representative scanning electron microscope image (5000× and 25000×) and (B) elemental composition analysis of (C) abraded versus non‐abraded regions on surfaces treated with a Ti curette. The stacked bar chart shows the relative abundance of carbon (C), oxygen (O), Ti, and other elements. Delaminated, abraded regions on curette‐treated surfaces demonstrated a slight decrease in the percentage of O compared to the non‐abraded regions. Ti, titanium.

Representative SEM images for each treatment group are shown in Figure 3. Untreated disks presented a robust and thick biofilm (Figure 3, upper right panel). Disks treated with Ti curette demonstrated scratch marks and delaminated layers of Ti surface (red arrowheads in Figure 3, middle right panel). These features were also present to a lesser extent on Ti brush‐treated samples (Figure 3, lower right panel) suggesting surface micro‐alterations to the titanium oxide layer by the Ti brush. In contrast, disks cleaned by waterjet irrigation (Figure 3, middle and lower left panels) did not demonstrate any apparent changes in surface morphology as compared to uncontaminated pristine control (Figure 3, upper left panel). Also, scattered residual microbes were more abundant on the surface of curette‐ or brush‐treated Ti disks versus waterjet‐treated Ti disks. EDS analysis revealed a reduction in atomic oxygen (O) in the scratched titanium layers (Figure 4B, C), which demonstrated a “melted metal” appearance after abrasion as compared to the non‐scratched areas (Figure 4A). This reduction in oxygen suggests either passivation layer removal and exposure of titanium metal, or significant abrasion and thinning of the passivation layer based on the depth of the EDS profiling.

#### 3.2.2 Surface roughness analysis post‐treatment

**FIGURE 5:**
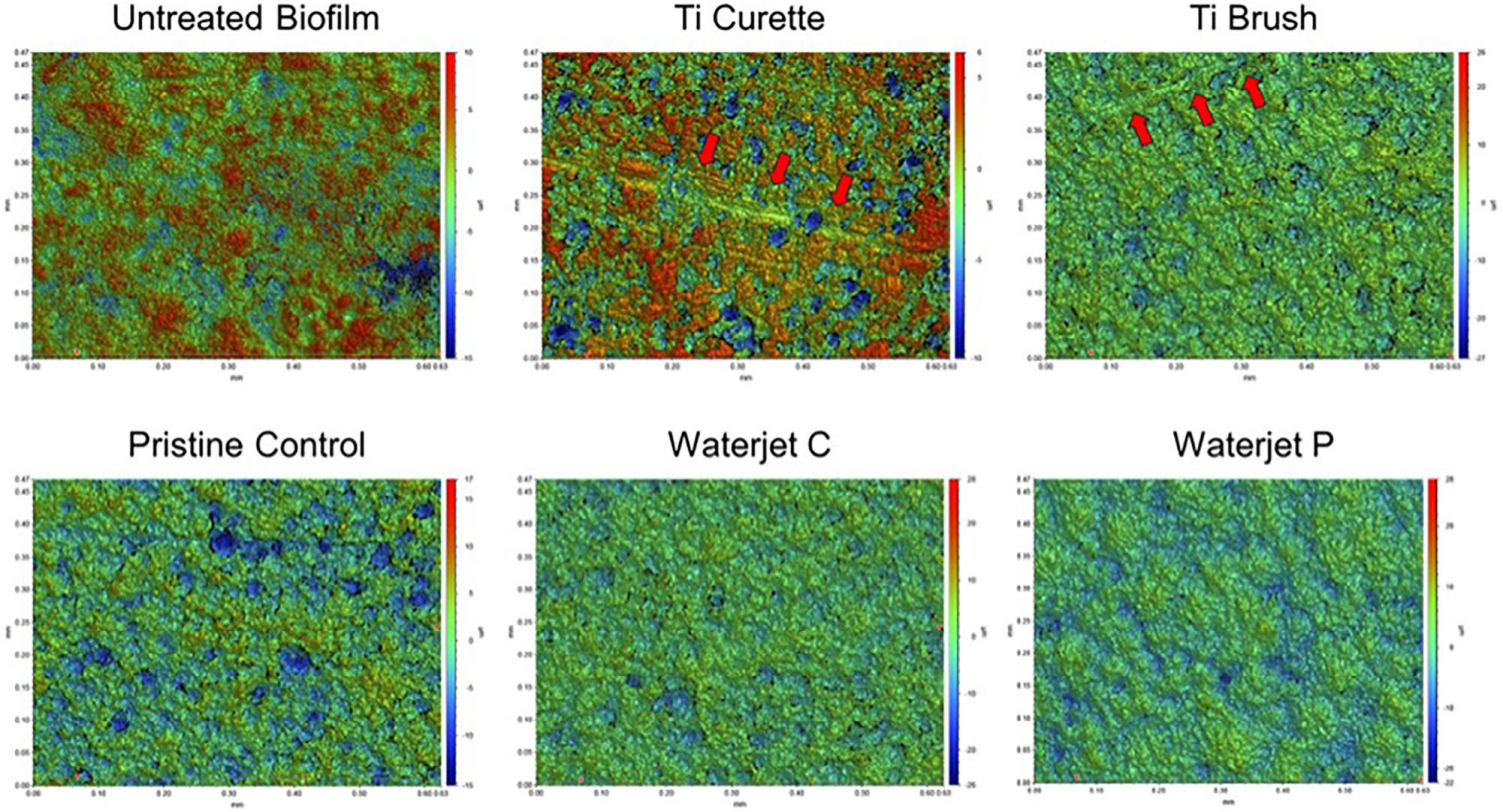
Representative surface roughness maps obtained using 3D optical profilometry of Ti disks after various cleaning treatments: Ti curette, Ti brush, waterjet continuous (C), waterjet pulse (P), negative control (pristine), and positive control (untreated biofilm) for biofilm removal. Color intensity represents the height of the surface features, where warmer colors (red/orange) indicate peaks and cooler colors (blue/green) indicate valleys. Surface abrasion (red arrows) was observed on Ti curette‐ and Ti brush‐treated samples. Ti, titanium.

Although no significant difference in average height (Ra) was observed across all sample groups (Figure S4 in online *Journal of Periodontology*), differences in surface microroughness profile between sample groups were evident as depicted in Figure 5. Greater coverage area of warmer colors (red/orange) were observed on the untreated biofilm and abraded areas (red arrows) of Ti curette‐treated samples indicating pronounced surface peaks due to residual biofilm, while greater coverage of cooler colors (blue/green) characteristic of the acid‐etched pristine control surface were also observed for Ti brush‐ and waterjet‐treated samples. Notably, both Ti‐curette and Ti‐brush treated samples showed signs of surface abrasion damage (red arrows in Figure 5) due to the applied cleaning treatment.

### 3.3 Cytocompatibility of treated titanium surfaces

**FIGURE 6:**
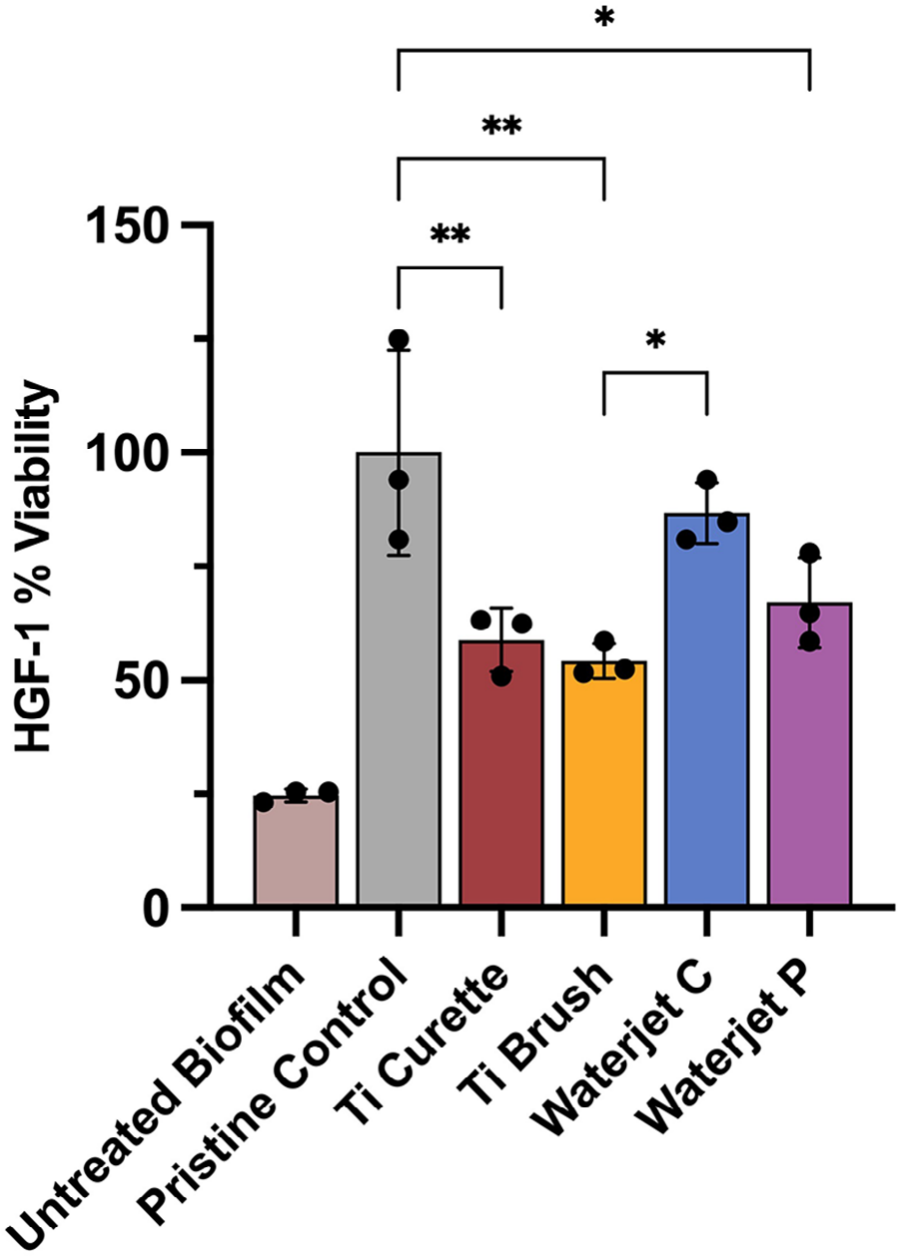
Fibroblast viability grown on Ti sample disks after treated with Ti curette, Ti brush, Waterjet Continuous continuous (C), or Waterjet Pulse pulse (P) cleaning versus untreated biofilm and pristine controls. The highest fibroblast count was observed in the Waterjet C treatment group, followed by Waterjet P, both significantly higher than the control and test groups (*p* < 0.05). Fibroblast viability (%) was calculated as the cell count of each group relative to the pristine control. Ti, titanium. ^*^ *p* < 0.05; ^**^ *p* < 0.001

To evaluate the cytocompatibility of Ti surfaces after cleaning treatment, human gingival fibroblasts were cultured on treated Ti disks following sterilization. A quantitative assessment of cell proliferation based on viable fibroblast cell count is shown in Figure 6. Among the test groups, Ti surface treated by waterjet cleaning with continuous flow (Waterjet C) had the highest fibroblast viability among all groups. Notably, the fibroblast proliferation on the Waterjet C surfaces was not significantly different than that of the positive pristine control (*p* > 0.05) suggesting restoration of cytocompatibility. The second‐best group was the pulsed waterjet cleaning (Waterjet P). Fewer viable fibroblasts were enumerated on Ti brush‐ and Ti curette‐treated samples, which were significantly lower than positive control (*p* < 0.05). The lowest fibroblast viability was observed on untreated biofilm surface.

## 4 DISCUSSION

Peri‐implantitis manifests as chronic inflammation in the tissues around the implant with progressive loss of supporting bone.^4^ Removal of microbial biofilm is a necessary but not sufficient component of peri‐implantitis treatments.^26^ Because of the established cytotoxic and pro‐inflammatory effects of implant degradation products,^27^, ^28^, ^29^ the preservation of the implant surface integrity for soft tissue seal formation and re‐osseointegration with bone has been recognized as a critical step to peri‐implantitis treatment.^30^ Adverse surface changes imparted during implant cleaning can hinder the regeneration of soft and hard tissue on the implant surface and even encourage bacterial recolonization.^13^, ^31^ Conventional methods of decontamination may inadvertently damage the implant surface, thus compromising its integrity and biocompatibility. In the present study, a non‐contact cleaning aid prototype machine based on pressurized irrigation was tested against conventional abrasive contact cleaning methods to remove biofilm from rough Ti surfaces. Grade 5 titanium alloy (Ti‐6Al‐4 V) disks were tested due to potentially increased susceptibility to corrosion than commercially pure titanium due to the presence of alloying elements.

There are multiple contact and non‐contact decontamination methods of implant cleaning; however, there is still no agreement on which technique is the most effective.^32^ Clinicians often employ aggressive interventions that directly contact the implant surface, such as implantoplasty, over non‐contact methods of implant decontamination, such as pressurized irrigation. This is due to the misconception that more abrasive contact methods are more capable of detaching biofilm and removing bacterial endotoxin.^24^ However, human evidence suggests that these abrasive efforts do not benefit implant survival with any clinical significance.^33^ To test the efficacy of the waterjet cleaning treatment in removing biofilm, a multispecies biofilm cultivated from disease‐associated plaque was grown in SHI medium per previous studies.^13^, ^25^ Propagation of plaque‐derived culture in SHI medium was shown to maintain several dozen genera of oral bacteria via 16S rRNA sequencing, especially gram‐negative pathogens including *Fusobacterium*, *Porphyromonas*, and *Tannerella* spp. associated with peri‐implantitis, and resulted in robust biofilm formation.^25^

Beginning with assessment of biofilm removal capacity, as shown in Figures 1 and 2, bacterial biofilm was removed to the greatest extent by waterjet cleaning, followed by Ti curette and Ti brush versus untreated biofilm control. Viable bacterial counts decreased significantly for all treatments versus control (*p* < 0.05), with the pressurized irrigation treatment reducing bacterial load to the lowest extent and up to ∼100 times more than mechanical abrasion by Ti curette and Ti brush (Figure 1). In a recent study by Stein et al., application of Ti curette on Ti surface did not show any significantly reduction in bacterial residue (rRNA count representing bacterial activity) as compared to the untreated control.^34^ Correspondingly, confocal imaging of waterjet‐treated Ti surface demonstrated the lowest amount of live and dead bacteria remaining with only isolated specks sparsely visible. In contrast, clusters of viable bacteria were visible within pockets on the non‐abraded regions of Ti surface treated by the Ti curette or Ti brush. Similarly, biofilm mass on Ti decreased after waterjet treatment and to a lesser extent with Ti curette or brush (Figure 3). These findings align with prior research showing that, although abrasive contact tools such as Ti brushes have a strong mechanical action,^35^ their antimicrobial effect is less than the non‐contact irrigation method. Similarly, the application of another waterjet irrigation resulted in almost complete biofilm removal from the Ti surface.^13^

In our study, surface roughness resembling the dental implant surface was achieved by acid etching. Surface roughness and wettability imparted by surface treatment are regarded as key factors affecting the initial bacterial accumulation, mechanical performance, corrosion resistance, and osseointegration of dental implants.^36^, ^37^, ^38^, ^39^, ^40^ Although mechanical abrasive contact treatment and pressurized irrigation by the waterjet system both reduced bacterial load, waterjet cleaning was superior in maintaining Ti surface condition. As shown in Figures 3 and 5, the Ti surface topography and 3D profile were best maintained with Bacterator treatment and appeared most similar to the untreated negative control surface. That is, the pressurized water irrigation surface demonstrated a rough surface appearance similar to control with almost no residual viable or dead bacteria present (Figure 3) and roughness nearly identical to untreated negative control (Figure 5). In contrast, mechanical treatment with Ti curette severely damaged the rough Ti surface (Figure 5) and exhibited a surface roughness profile comparable to untreated control with pronounced peaks characteristic of biofilm growth, which demonstrated poor removal of biofilm even on abraded surface areas. Although cleaning with Ti brush more efficiently removed biofilm than Ti curette treatment based on the observed surface profile (Figure 5), abraded regions exhibited signs of severe delamination and surface damage (Figure 3). Sousa et al. similarly observed that Ti brush treatment of biofilm grown on Ti surface resulted in scratched and modified surface and residual debris.^35^ These deleterious alterations in surface morphology were also seen by Sahrmann et al. after a 2‐min application of either steel curettes or ultrasound cleaning.^41^

The EDS elemental analysis revealed that titanium, oxygen, and carbon were the primary elements present on all treated disks (Figure 4). Small amounts of vanadium were detected on the surface, which aligns with the grade 5 titanium alloy (Ti‐6Al‐4 V) composition. On Ti curette‐treated samples, the abraded areas had lower average oxygen levels in the surface layer versus non‐abraded areas (Figure 4). This observed reduction in oxygen level may imply either the removal of the protective passivation layer, exposing the underlying titanium metal, or significant abrasion and thinning of the oxide layer as suggested by the depth of the EDS profiling. Specifically, the lower oxygen content on the scratched surfaces of disks treated with the Ti curette could indicate that abrasive treatment led to the loss of the oxide layer. It is now known that abrasion and loss of titanium passivation can lead to decreased corrosion resistance and, only under inflammatory conditions, to the formation of less stable oxides such as Ti_2_O_3_ and TiO.^21^ This partial repassivation with unstable oxides can have detrimental effects on long‐term surface integrity and titanium release.^21^ Ichioka et al. reported a significant decrease in the carbon composition of implant surfaces after decontamination with Ti brush but also notable surface alterations.^42^ They also stated that no clear advantage was demonstrated for any of the mechanical or chemical methods used such as air‐polishing, rotating Ti brush, or alkaline electrolyzed water.^42^

Although previous studies have shown that smoothening of the surface reduces the chance for biofilm reformation,^43^, ^44^ this surface also hinders the potential for tissue attachment and re‐osseointegration.^45^, ^46^, ^47^ In the present study, abrasive mechanical treatment by Ti curette or Ti brush resulted in 41.4% and 45.8% reduction in viable fibroblast count, respectively, versus pristine control (Figure 6). In contrast, continuous mode waterjet cleaning had fibroblast cell count similar to that of pristine control (86.6%). Overall, contact abrasive mechanical cleaning by Ti curette or Ti brush was only moderately effective in eradicating biofilm on the Ti surface while adversely altering the surface morphology of titanium implants and impeding fibroblast growth. In contrast, waterjet cleaning significantly reduced viable bacteria and biofilm to the greatest extent on Ti surface with minimal surface alterations and maintained fibroblast viability similar to pristine control samples.

The presence of Ti particles is associated with recurring peri‐implant disease,^48^ and previous studies have demonstrated the cytotoxicity of Ti wear particles.^20^, ^49^ Implant‐derived Ti particles (iTiPs) are found to be more abundant under inflammatory conditions compared to relative clinical health, suggesting their involvement in peri‐implant inflammation.^50^ Titanium nanoparticles have been shown to upregulate the release of cytokines such as interleukin (IL)‐1β and increase bone resorption by inhibiting osteoblast proliferation and inducing osteoclast activity.^49^ The metallic wear debris also has been demonstrated to increase reactive oxygen species (ROS) generation which can lead to mitochondrial dysfunction and cell apoptosis and endanger bone formation around dental implants.^51^ A previous study showed 15% higher murine fibroblast cellular death as a result of stimulation with aged titanium dissolution products.^13^ Also, surface decontamination with waterjet irrigation demonstrated significantly higher fibroblast viability compared to the controls, while Ti brush‐treated disks had notably fewer viable cells.^13^ This is in accordance with our results indicating the cytotoxic effects of surface alterations and iTiPs on the regrowth of cells after treatment.

Although the present study demonstrated the promising potential of waterjet irrigation cleaning, several limitations must be addressed. Dental implant cleaning was performed with flat, disk‐shaped specimens under open‐air test conditions. However, the clinical scenario is more challenging due to the complex implant macro‐geometry providing less accessible surface areas for implant cleaning, such as within crevices between modular implant components and subgingival implant surfaces. Nonetheless, use of the same waterjet non‐contact irrigation for cleaning has led to safe and effective application in vivo in canines with actual microrough implants, suggesting in vivo validation of the presented results.^52^ In the present study, multispecies culture was grown for only 48 h, resulting in an adherent yet unmatured biofilm, which does not represent mature clinical peri‐implantitis biofilms. Regarding surface integrity analysis, only average surface roughness, Ra, was investigated. Future studies will assess changes in other surface roughness parameters and at the nanoscale in addition to quantitative measure of titanium particle release from treated samples after implant cleaning treatment. Moreover, cytocompatibility with host tissue was only assessed with human gingival fibroblasts representing soft tissue. A follow‐up mechanistic study will assess a novel repassivation strategy using osteoblasts to assess the potential for re‐osseointegration with treated titanium surface.

## 5 CONCLUSIONS

In conclusion, our study supports the growing body of evidence demonstrating waterjet cleaning as an effective approach to treat peri‐implantitis by reducing implant biofilms while preserving implant surface integrity and promoting cytocompatibility with host tissue. While conventional implant cleaning employs abrasive contact methods like titanium brushes and curettes for implant decontamination, waterjet irrigation provides a more robust and comprehensive implant cleaning method by efficiently detaching bacterial biofilm while maintaining the native Ti surface, thereby promoting compatibility and reintegration with host tissue. Within the limitations of the present study, waterjet irrigation is a promising implant cleaning method that meets the standards for peri‐implantitis treatment by providing superior biofilm removal capacity while promoting titanium surface integrity and compatibility with host tissue cells, thereby supporting long‐term implant success.

## AUTHOR CONTRIBUTIONS

All authors have made substantial contributions to this study. Marzieh S. Jazaeri, Danyal A. Siddiqui, Yi‐Wen C. Tsai, Kathryn Gabel, and Zachary Lorenzana were involved in the process of data collection and data analysis. Marzieh S. Jazaeri, Danyal A. Siddiqui, and Georgios A. Kotsakis have been involved in data interpretation, drafting the manuscript, and revising it critically. Marzieh S. Jazaeri and Danyal A. Siddiqui equally contributed to the study and are co‐first authors. All authors have given final approval for the version to be published.

## CONFLICT OF INTEREST STATEMENT

Dr. Kotsakis is a co‐inventor in a patent application related to DENTAL IRRIGATOR AND METHOD OF USE. Other authors declared no conflicts of interest related to this study.

## Supporting information



Supporting Information

Supporting Information

Supporting Information

Supporting Information

Supporting Information

## Data Availability

The data that support the findings of this study are available from the corresponding author upon reasonable request.

## Footnotes

* BT917, Benchtop Lab Systems, Burlington, VT, USA

† WPA CO8000, Biochrom Ltd., Cambridge, United Kingdom

‡ STERLING Supreme Resin Handle Titanium Implant Scaler, Sterling Dental Instruments, Lemoyne, PA, USA

§ RotoBrush‐Titanium Thread Cleaning Brush, Salvin Dental Specialties, Charlotte, NC, USA

‖ Bacterator, ImplantSci Ltd., Cyprus

¶ Leica Stellaris 8 tau‐STED Super Resolution Microscope

# Infinite Pro F200, Tecan Group Ltd., Männedorf, Zürich, Switzerland

** Phenom SEM ProX, NanoScience Instruments, Inc., Phoenix, AZ, USA

†† Contour GT, Bruker Nano Surfaces Division, Tucson, AZ, USA

‡‡ ATCC, Manassas, VA, USA

§§ NucBlue Fixed Cell ReadyProbes Reagent, Invitrogen, Thermo Fisher Scientific, Carlsbad, CA, USA

‖‖ ActinGreen 488 ReadyProbes Reagent, Invitrogen, Thermo Fisher Scientific, Carlsbad, CA, USA

¶¶ ECLIPSE Ts2R, Nikon Instruments, Melville, NY, USA

## Image J, National Institutes of Health, Bethesda, MD, USA

*** GraphPad Prism, version 10.2.3, Boston, MA, USA

## References

[1] Research Nester . Peri‐implantitis treatment market size & share, statistics report 2024‐2036 . 2024. Accessed: Sep 13, 2024. Available at: https://www.researchnester.com/reports/peri‐implantitis‐treatment‐market/3922

[2] Diaz P , Gonzalo E , Villagra LJG , et al. What is the prevalence of peri‐implantitis? A systematic review and meta‐analysis. BMC Oral Health. 2022;22:449. doi:10.1186/s12903-022-02493-8 36261829 PMC9583568

[3] American Academy of Implant Dentistry . Trends in dental implants 2022 . 2024. Accessed: Sep 13, 2024. Available at: https://connect.aaid‐implant.org/blog/trends‐in‐dental‐implants‐2022

[4] Schwarz F , Derks J , Monje A , et al. Peri‐implantitis. J Clin Periodontol. 2018;45:S246‐S266. doi:10.1111/jcpe.12954 29926484

[5] Kensara A , Saito H , Mongodin EF , et al. Microbiological profile of peri‐implantitis: analyses of microbiome within dental implants. J Prosthodont. 2023;32:783‐792. doi:10.1111/jopr.13653 36691777

[6] Belibasakis GN , Charalampakis G , Bostanci N . Peri‐implant infections of oral biofilm etiology. In: Donelli G , ed. Biofilm‐Based Healthcare‐Associated Infections: Volume I. Springer International Publishing; 2015:69‐84. doi:10.1007/978-3-319-11038-7_4 25366221

[7] Rokaya D , Srimaneepong V , Wisitrasameewon W , et al. Peri‐implantitis update: risk indicators, diagnosis, and treatment. Eur J Dent. 2020;14:672‐682. doi:10.1055/s-0040-1715779 32882741 PMC7536094

[8] Toma S , Brecx MC , Lasserre JF . Clinical evaluation of three surgical modalities in the treatment of peri‐implantitis: a randomized controlled clinical trial. J Clin Med. 2019;8:966. doi:10.3390/jcm8070966 31277265 PMC6679014

[9] de Carvalho GG , Sanchez‐Puetate JC , Casalle N , et al. Antimicrobial photodynamic therapy associated with bone regeneration for peri‐implantitis treatment: a case report. Photodiagnosis Photodyn Ther. 2020;30:101705. doi:10.1016/j.pdpdt.2020.101705 32135313

[10] Stiesch M , Grischke J , Schaefer P , et al. Supportive care for the prevention of disease recurrence/progression following peri‐implantitis treatment: a systematic review. J Clin Periodontol. 2023;50(Suppl 26):113‐134. doi:10.1111/jcpe.13822 37339881

[11] Ronay V , Merlini A , Attin T , et al. In vitro cleaning potential of three implant debridement methods. Simulation of the non‐surgical approach. Clin Oral Implants Res. 2017;28:151‐155. doi:10.1111/clr.12773 26799360

[12] Barrak FN , Li S , Muntane AM , et al. Particle release from implantoplasty of dental implants and impact on cells. Int J Implant Dent. 2020;6:50. doi:10.1186/s40729-020-00247-1 32918144 PMC7486360

[13] Kotsakis GA , Black R , Kum J , et al. Effect of implant cleaning on titanium particle dissolution and cytocompatibility. J Periodontol. 2021;92:580‐591. doi:10.1002/jper.20-0186 32846000

[14] Souza JGS , Costa Oliveira BE , Bertolini M , et al. Titanium particles and ions favor dysbiosis in oral biofilms. J Periodontal Res. 2020;55:258‐266. doi:10.1111/jre.12711 31762055

[15] Asa'ad F , Thomsen P , Kunrath MF . The role of titanium particles and ions in the pathogenesis of peri‐implantitis. J Bone Metab. 2022;29:145‐154. doi:10.11005/jbm.2022.29.3.145 36153850 PMC9511127

[16] Wang X , Li Y , Feng Y , et al. Macrophage polarization in aseptic bone resorption around dental implants induced by ti particles in a murine model. J Periodontal Res. 2019;54:329‐338. doi:10.1111/jre.12633 30635919

[17] Stolzer C , Müller M , Gosau M , et al. Do titanium dioxide particles stimulate macrophages to release proinflammatory cytokines and increase the risk for peri‐implantitis?. J Oral Maxillofac Surg. 2023;81:308‐317. doi:10.1016/j.joms.2022.10.019 36442535

[18] Barrak FN , Li S . From manufacturers to clinicians, the release of dental implant particles can no longer be ignored. Clin Implant Dent Relat Res. 2024;26:663‐667. doi:10.1111/cid.13309 38369955

[19] Kandaswamy E , Harsha M , Joshi VM . Titanium corrosion products from dental implants and their effect on cells and cytokine release: a review. J Trace Elem Med Biol. 2024;84:127464. doi:10.1016/j.jtemb.2024.127464 38703537

[20] Callejas JA , Gil J , Brizuela A , et al. Effect of the size of titanium particles released from dental implants on immunological response. Int J Mol Sci. 2022;23:7333. 35806339 10.3390/ijms23137333 PMC9266706

[21] Kotsakis GA , Xie L , Siddiqui DA , et al. Dynamic assessment of titanium surface oxides following mechanical damage reveals only partial passivation under inflammatory conditions. NPJ Mater Degrad. 2024;8:98. doi:10.1038/s41529-024-00514-1 39583186 PMC11578884

[22] Sousa V , Mardas N , Spratt D , et al. Experimental models for contamination of titanium surfaces and disinfection protocols. Clin Oral Implants Res. 2016;27:1233‐1242. doi:10.1111/clr.12735 26864128

[23] Bassetti M , Bassetti R , Sculean A , et al. [subcutaneous emphysema following non‐surgical peri‐implantitis therapy using an air abrasive device: a case report]. Swiss Dent J. 2014;124:807‐817. doi:10.61872/sdj-2014-07-08-04 25118639

[24] Monje A , Amerio E , Cha JK , et al. Strategies for implant surface decontamination in peri‐implantitis therapy. Int J Oral Implantol. 2022;15:213‐248. 36082658

[25] Lamont EI , Gadkari A , Kerns KA , et al. Modified shi medium supports growth of a disease‐state subgingival polymicrobial community in vitro. Mol Oral Microbiol. 2021;36:37‐49. doi:10.1111/omi.12323 33174294 PMC7984074

[26] Schwarz F , Papanicolau P , Rothamel D , et al. Influence of plaque biofilm removal on reestablishment of the biocompatibility of contaminated titanium surfaces. J Biomed Mater Res A. 2006;77:437‐444. doi:10.1002/jbm.a.30628 16444683

[27] Schwarz F , Langer M , Hagena T , et al. Cytotoxicity and proinflammatory effects of titanium and zirconia particles. Int J Implant Dent. 2019;5:25. doi:10.1186/s40729-019-0178-2 31286286 PMC6614223

[28] Pettersson M , Kelk P , Belibasakis GN , et al. Titanium ions form particles that activate and execute interleukin‐1β release from lipopolysaccharide‐primed macrophages. J Periodontal Res. 2017;52:21‐32. doi:10.1111/jre.12364 26987886 PMC5297875

[29] Wakuda S , Hasuike A , Fujiwara K , et al. Titanium particle‐induced inflammasome in human gingival epithelial cells. J Dent Sci. 2025;20:384‐392. doi:10.1016/j.jds.2024.06.013 39873089 PMC11762583

[30] Wilson TG Jr . Bone loss around implants‐is it metallosis?. J Periodontol. 2021;92:181‐185. doi:10.1002/jper.20-0208 32729118

[31] Padulles‐Gaspar E , Padulles‐Roig E , Cabanes G , et al. Effects of hypochlorous acid and hydrogen peroxide treatment on bacterial disinfection treatments in implantoplasty procedures. Materials. 2023;16:2953. doi:10.3390/ma16082953 37109795 PMC10144543

[32] Francis S , Iaculli F , Perrotti V , et al. Titanium surface decontamination: a systematic review of in vitro comparative studies. Int J Oral Maxillofac Implants. 2022;37:76‐84. doi:10.11607/jomi.8969 35235623

[33] Ravidà A , Siqueira R , Saleh I , et al. Lack of clinical benefit of implantoplasty to improve implant survival rate. J Dent Res. 2020;99:1348‐1355. doi:10.1177/0022034520944158 32718212

[34] Stein JM , Conrads G , Abdelbary MMH , et al. Antimicrobial efficiency and cytocompatibility of different decontamination methods on titanium and zirconium surfaces. Clin Oral Implants Res. 2023;34:20‐32. doi:10.1111/clr.14014 36259118

[35] Sousa V , Mardas N , Spratt D , et al. The effect of microcosm biofilm decontamination on surface topography, chemistry, and biocompatibility dynamics of implant titanium surfaces. Int J Mol Sci. 2022;23:10033. doi:10.3390/ijms231710033 36077428 PMC9456268

[36] Montero J , Fernández‐Ruiz A , Pardal‐Peláez B , et al. Effect of rough surface platforms on the mucosal attachment and the marginal bone loss of implants: a dog study. Materials. 2020;13:802. doi:10.3390/ma13030802 32050603 PMC7040816

[37] London RM , Roberts FA , Baker DA , et al. Histologic comparison of a thermal dual‐etched implant surface to machined, tps, and ha surfaces: bone contact in vivo in rabbits. Int J Oral Maxillofac Implants. 2002;17:369‐376. 12074452

[38] Matos GRM . Surface roughness of dental implant and osseointegration. J Maxillofac Oral Surg. 2021;20:1‐4. doi:10.1007/s12663-020-01437-5 33584035 PMC7855123

[39] Wassmann T , Kreis S , Behr M , et al. The influence of surface texture and wettability on initial bacterial adhesion on titanium and zirconium oxide dental implants. Int J Implant Dent. 2017;3:32. doi:10.1186/s40729-017-0093-3 28714053 PMC5511811

[40] Sridhar S , Abidi Z . In vitro evaluation of the effects of multiple oral factors on dental implants surfaces. J Oral Implantol. 2016;42:248‐257. doi:10.1563/aaid-joi-D-15-00165 26829492

[41] Sahrmann P , Ronay V , Hofer D , et al. In vitro cleaning potential of three different implant debridement methods. Clin Oral Implants Res. 2015;26:314‐319. doi:10.1111/clr.12322 24373056

[42] Ichioka Y , Virto L , Nuevo P , et al. Decontamination of biofilm‐contaminated implant surfaces: an in vitro evaluation. Clin Oral Implants Res. 2023;34:1058‐1072. doi:10.1111/clr.14136 37469250

[43] Bermejo P , Sánchez MC , Llama‐Palacios A , et al. Biofilm formation on dental implants with different surface micro‐topography: an in vitro study. Clin Oral Implants Res. 2019;30:725‐734. doi:10.1111/clr.13455 31077449

[44] Choi S , Jo Y‐H , Luke Yeo I‐S , et al. The effect of surface material, roughness and wettability on the adhesion and proliferation of *streptococcus gordonii*, *fusobacterium nucleatum* and *porphyromonas gingivalis* . J Dent Sci. 2023;18:517‐525. doi:10.1016/j.jds.2022.09.010 37123448 PMC10131180

[45] Le Guéhennec L , Soueidan A , Layrolle P , et al. Surface treatments of titanium dental implants for rapid osseointegration. Dent Mater. 2007;23:844‐854. doi:10.1016/j.dental.2006.06.025 16904738

[46] Park JY , Davies JE . Red blood cell and platelet interactions with titanium implant surfaces. Clin Oral Implants Res. 2000;11:530‐539. doi:10.1034/j.1600-0501.2000.011006530.x 11168246

[47] Vlacic‐Zischke J , Hamlet SM , Friis T , et al. The influence of surface microroughness and hydrophilicity of titanium on the up‐regulation of tgfβ/bmp signalling in osteoblasts. Biomaterials. 2011;32:665‐671. doi:10.1016/j.biomaterials.2010.09.025 20933273

[48] Daubert D , Lee E , Botto A , et al. Assessment of titanium release following non‐surgical peri‐implantitis treatment: a randomized clinical trial. J Periodontol. 2023;94:1122‐1132. doi:10.1002/JPER.22-0716 37070363 PMC10524263

[49] Messous R , Henriques B , Bousbaa H , et al. Cytotoxic effects of submicron‐ and nano‐scale titanium debris released from dental implants: an integrative review. Clin Oral Investig. 2021;25:1627‐1640. doi:10.1007/s00784-021-03785-z 33616805

[50] Olmedo DG , Nalli G , Verdú S , et al. Exfoliative cytology and titanium dental implants: a pilot study. J Periodontol. 2013;84:78‐83. doi:10.1902/jop.2012.110757 22414261

[51] Yang F , Tang J , Dai K , et al. Metallic wear debris collected from patients induces apoptosis in rat primary osteoblasts via reactive oxygen species‑mediated mitochondrial dysfunction and endoplasmic reticulum stress. Mol Med Rep. 2019;19:1629‐1637. doi:10.3892/mmr.2019.9825 30628694 PMC6390047

[52] Esplin KC , Tsai YW , Vela K , et al. Peri‐implantitis induction and resolution around zirconia versus titanium implants. J Periodontol. 2024;95:1180‐1189. doi:10.1002/jper.23-0573 39003566 PMC11708443

