# Supplementary material for: Biofilm removal capacity and titanium surface integrity in non‐abrasive versus abrasive peri‐implantitis cleaning interventions
Source: J Periodontol. 2025 Dec 10;97(3):498–510. doi: 10.1002/jper.11371 (PMC13111778; doi:10.1002/jper.11371)
Supplement: Supplementary file 1 — Supporting Information [file JPER-97-498-s003.docx]

**
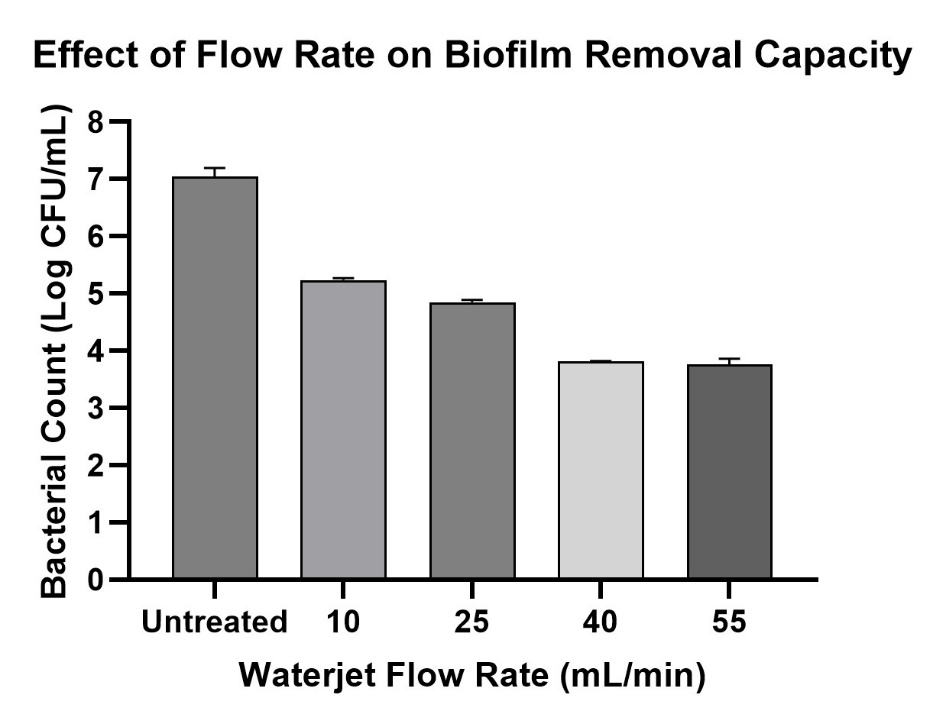
**

**Figure S1.** Bacterial count (log CFU/mL) of residual biofilm after application of waterjet irrigation at different flow rates (10, 25, 40, 55, 70, and 85 mL/min) versus untreated control.
