# Supplementary material for: Biofilm removal capacity and titanium surface integrity in non‐abrasive versus abrasive peri‐implantitis cleaning interventions
Source: J Periodontol. 2025 Dec 10;97(3):498–510. doi: 10.1002/jper.11371 (PMC13111778; doi:10.1002/jper.11371)
Supplement: Supplementary file 2 — Supporting Information [file JPER-97-498-s001.docx]

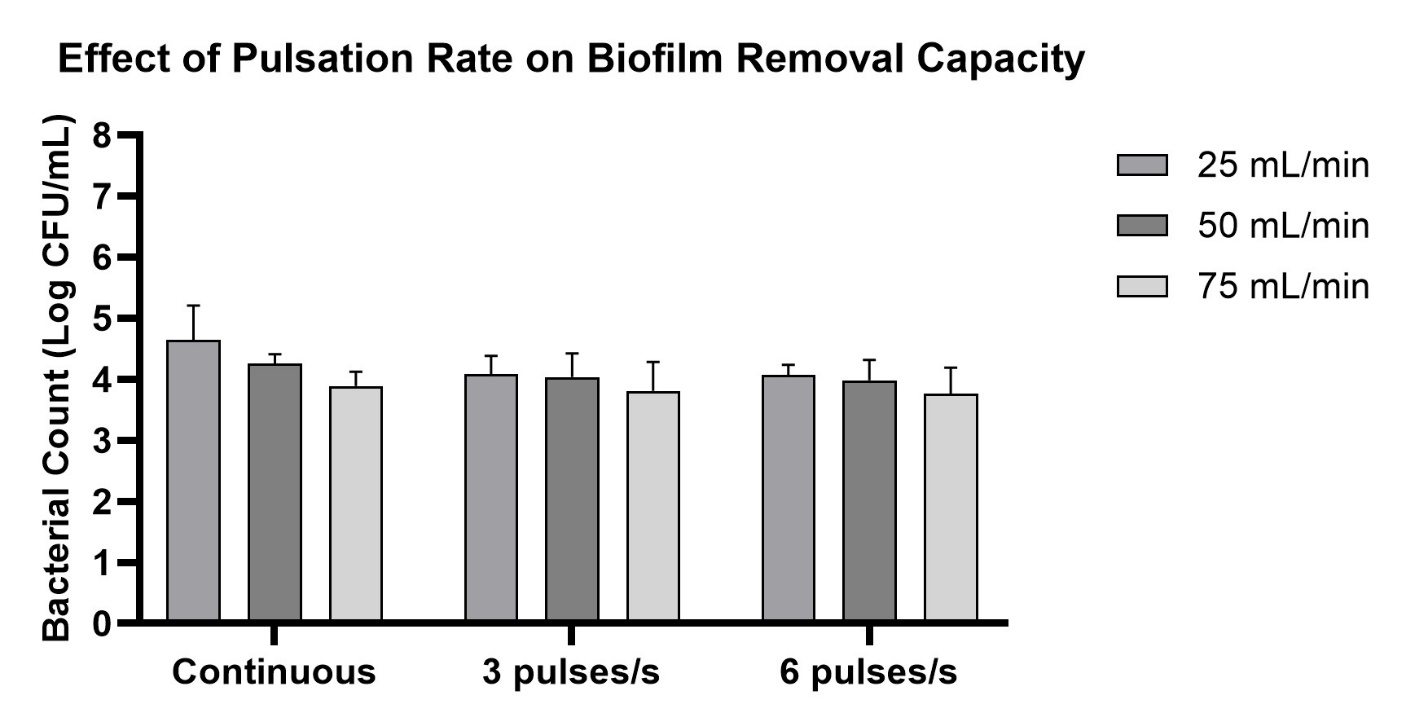


**Figure S2.** Bacterial count (log CFU/mL) of residual biofilm after application of waterjet irrigation at different flow (25, 50, 75 mL/min) and pulsation rates (continuous, 0, and 3 pulses/s).
