# Supplementary material for: Biofilm removal capacity and titanium surface integrity in non‐abrasive versus abrasive peri‐implantitis cleaning interventions
Source: J Periodontol. 2025 Dec 10;97(3):498–510. doi: 10.1002/jper.11371 (PMC13111778; doi:10.1002/jper.11371)
Supplement: Supplementary file 3 — Supporting Information [file JPER-97-498-s002.docx]

**
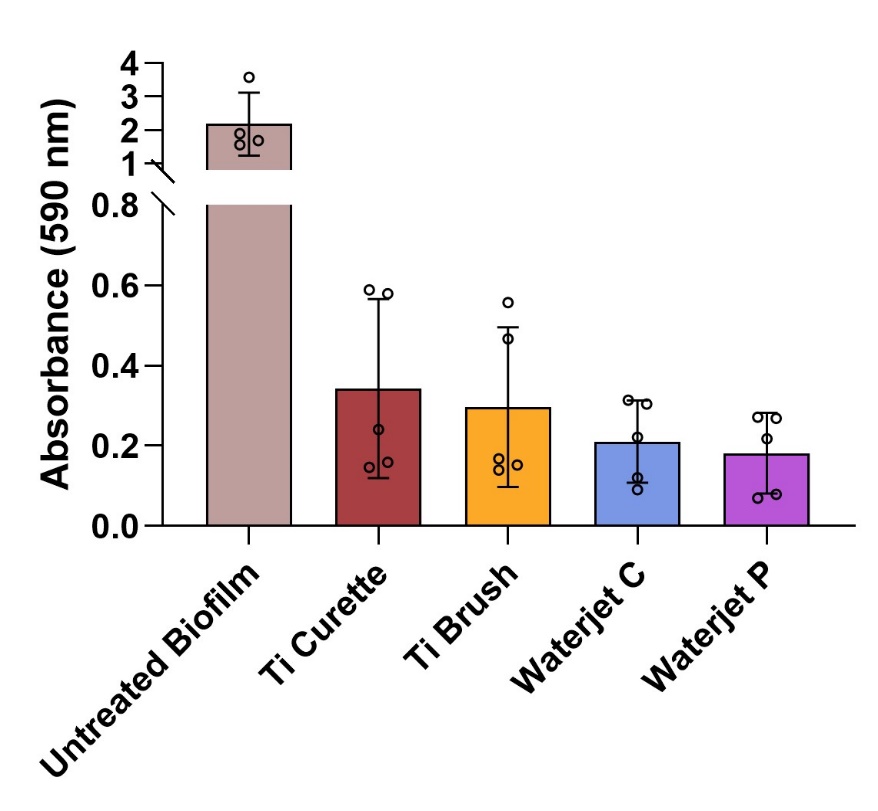
**

**Figure S3.** Absorbance readings (OD590) of residual crystal violet-stained biofilms on Ti disks after various cleaning treatments: Ti curette, Ti brush, waterjet continuous (C), waterjet pulse (P), negative control (pristine) and positive control (untreated biofilm) for biofilm removal.
