# Supplementary material for: Biofilm removal capacity and titanium surface integrity in non‐abrasive versus abrasive peri‐implantitis cleaning interventions
Source: J Periodontol. 2025 Dec 10;97(3):498–510. doi: 10.1002/jper.11371 (PMC13111778; doi:10.1002/jper.11371)
Supplement: Supplementary file 5 — Supporting Information [file JPER-97-498-s005.docx]

**Table S1. Top 50 bacterial species present in plaque-derived multispecies culture.**

| **Genus** | **Species** |
| --- | --- |
| *Fusobacterium* | *nucleatum_subsp._animalis* |
| *Peptostreptococcus* | *stomatis* |
| *Solobacterium* | *moorei* |
| *Fusobacterium* | *nucleatum_subsp._vincentii* |
| *Prevotella* | *buccae* |
| *Parvimonas* | *sp._HMT_110* |
| *Porphyromonas* | *pasteri* |
| *Peptostreptococcaceae_[XI][G-1]* | *infirmum* |
| *Catonella* | *sp._HMT_164* |
| *Prevotella* | *nigrescens* |
| *Rothia* | *dentocariosa* |
| *Rothia* | *aeria* |
| *Erythromicrobium* | *ramosum* |
| *Peptostreptococcus* | *anaerobius* |
| *Corynebacterium* | *matruchotii* |
| *Stomatobaculum* | *longum* |
| *Alloprevotella* | *rava* |
| *Capnocytophaga* | *sputigena* |
| *Actinomyces* | *sp._HMT_169* |
| *Fusobacterium* | *nucleatum_subsp._polymorphum* |
| *Prevotella* | *sp._HMT_317* |
| *Saccharibacteria_(TM7)_[G-1]* | *bacterium_HMT_952* |
| *Atopobium* | *parvulum* |
| *Porphyromonas* | *sp._HMT_278* |
| *Clostridiales_[F-3][G-1]* | *bacterium_HMT_876* |
| *Porphyromonas* | *sp._HMT_275* |
| *Bifidobacterium* | *dentium* |
| *Campylobacter* | *concisus* |
| *Capnocytophaga* | *leadbetteri* |
| *Alloprevotella* | *sp._HMT_473* |
| *Leptotrichia* | *sp._HMT_215* |
| *Lachnoanaerobaculum* | *orale* |
| *Campylobacter* | *gracilis* |
| *Mogibacterium* | *neglectum* |
| *Eggerthia* | *catenaformis* |
| *Tannerella* | *forsythia* |
| *Prevotella* | *oris* |
| *Sphingomonas* | *echinoides* |
| *Brevundimonas* | *diminuta* |
| *Agrobacterium* | *tumefaciens* |
| *Porphyromonas* | *sp._HMT_930* |
| *Lachnospiraceae_[G-7]* | *bacterium_HMT_086* |
| *Lachnospiraceae_[G-2]* | *bacterium_HMT_088* |
| *Microbacterium* | *flavescens* |
| *Lachnospiraceae_[G-2]* | *bacterium_HMT_096* |
| *Oribacterium* | *asaccharolyticum* |
| *Bacteroides* | *heparinolyticus* |
| *Pedobacter* | *sp._HMT_933* |
| *Fastidiosipila* | *sanguinis* |
| *Capnocytophaga* | *sp._HMT_336* |
